# Supplementary material for: The interactome of intact mitochondria by cross-linking mass spectrometry provides evidence for coexisting respiratory supercomplexes
Source: Mol Cell Proteomics. 2017 Dec 8;17(2):216–32. doi: 10.1074/mcp.RA117.000470 (PMC5795388; doi:10.1074/mcp.RA117.000470)
Supplement: Supplemental Data [file supp_RA117.000470_133922_0_supp_23978_fzffwf.zip › spectra_annotation/mito_DR_spectra_annotation/29-1-9-1-5-1.pdf]

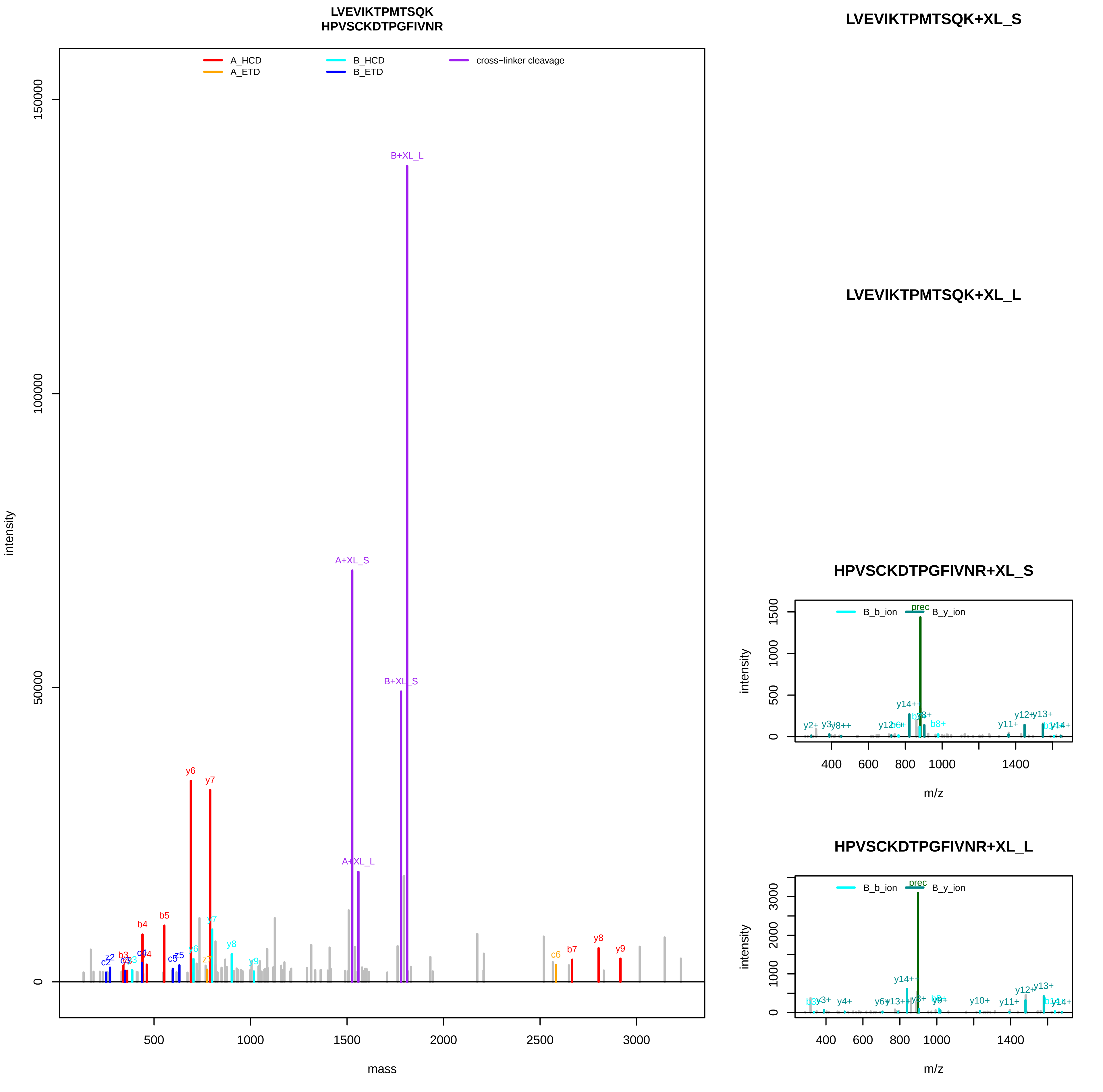

LVEVIKTPMTSQK
HPVSCKDTPGFIVNR
A_HCD
A_ETD
B_HCD
B_ETD
cross−linker cleavage
B+XL_L
A+XL_S
B+XL_S
A+XL_L
y6
y7
b4
b5
y8
y9
b7
c6
b3
c2
z2
c3
c4
c5
z5
intensity
mass
0
50000
100000
150000
500
1000
1500
2000
2500
3000
LVEVIKTPMTSQK+XL_S
LVEVIKTPMTSQK+XL_L
HPVSCKDTPGFIVNR+XL_S
B_b_ion
B_y_ion
prec
y14++
y2+
y11+
y12+
y13+
b8+
intensity
m/z
0
500
1000
1500
400
600
800
1000
1400
HPVSCKDTPGFIVNR+XL_L
B_b_ion
B_y_ion
prec
y14++
y3+
y4+
y10+
y11+
y12+
y13+
y14+
intensity
m/z
0
1000
2000
3000
400
600
800
1000
1400
